# The potential of one-part alkali-activated materials (AAMs) as a concrete patch mortar

**DOI:** 10.1038/s41598-022-19830-0

**Published:** 2022-09-23

**Authors:** Eddy Yusslee, S. Beskhyroun

**Affiliations:** Auckland University of Technology (AUT), Auckland, 1010 New Zealand

**Keywords:** Environmental sciences, Engineering, Materials science

## Abstract

One-part alkali-activated materials (AAMs) are developed to improve conventional two-part systems. One-part AAMs technology has been used in cement binders to produce concrete, mortar, and paste. Current research mainly focuses on synthesizing raw materials obtained from industrial and agricultural waste as the main aluminosilicate precursors of the cement binder for a concrete application. The one-part AAMs were reported to have higher early compressive strength at 7 days of age, contributed by its fast-setting time, mainly when the binder activates by a higher dosage of alkaline activator and containing OPC-rich. Due to bonding issues, single or combination, FA/GGBFS/MK precursors were reported as unsuitable for use as a concrete repair material. They were the reason for the lack of one-part AAMs application of mortar compared to concrete usage. This study was conducted to determine the potential of one-part AAMs used as concrete patch mortar by investigating its rheology and mechanical properties. The compressive strength of the mortar was tested under lab ambient temperature in the tropical climate country of Malaysia. The setting time of fresh mortar and bonding strength were set under controlled lab temperature. The one-part alkali-activated mortar was composed of hybrid aluminosilicate precursors between fly ash (FA), Ground Granulated Blast Furnace Slag (GGBFS) and ordinary Portland cement (OPC). A low alkaline activator of solid potassium carbonate was used for the geopolymerization process. Three types of solid admixtures were added to complete the composition of the new mix design. The experiment's outcome showed that the mortar composed with the combination of conventional Portland cement and industrial waste products has compressive and pull-off adherence strength that meets with Class R3—EN1504-3 standard for structural concrete repair materials requirement.

## Introduction

Many concrete buildings are approaching or exceeding their designed service life and require substantial maintenance to ensure the structure can function to the end of its service life. Typically, concrete infrastructures are designed for 50 to 100 years or more. However, most concrete buildings were built in the past 50 years, where sustainable construction materials are not widely used and established yet. For economic reasons, building owners tend to repair and upgrade these ageing buildings rather than demolish them, and also from the ecstatic or historical point of view, which makes them want to keep the existing building^1^.

Concrete degradation is most common due to corrosion, cracks, spalling, etc., and is repaired with different types of concrete repair techniques and strategies. Presently the maintenance program is focusing on developing eco-friendly materials such as geopolymer or other types of sustainable cement, which not only offers more environmentally friendly products but also improves its engineering properties like higher mechanical strength and better microstructure compared to the traditional ordinary Portland cement (OPC), which extensively used.

Ordinary Portland cement (OPC) has been used as a concrete binder due to its good mechanical properties and cheapness, making it a popular choice in construction^2^. However, many researchers have started looking for alternative binders to replace the OPC due to the environmental impact. Alkali-activated materials (AAMs) are a new binder introduced in the market. It is known for its recycle-friendly products by promoting and utilizing industrial waste as the primary aluminosilicate precursor source. Lately, the conventional two-part AAMs system has been deliberately replaced by one-part technology, also referred to as the ‘just add water’ concept, where the binder is composed of solid precursors, activated by a solid alkali activator before water is added to activate the mixture, unlike the two-part method that still required corrosive aqueous solutions of alkali activator, not convenient for handling purpose from mixing, transporting to the placing of the sticky concrete. The one-part AAMs used as concrete proved similar and had better mechanical performance than the OPC.

On the contrary, AAMs applications as concrete repair materials are still not popular compared to commercial polymer-modified cement mortar and epoxy resin. Therefore, the effort has been made to ensure the mechanical strength compatibility will be better or comparable to the conventional concrete repair materials available in the market. AAMs, as part of geopolymer cementitious technology reported has better flexural bond strength than the OPC due to the creation of chemical bonds, especially C-S–H gels for interfacial bonding at the interfacial transition zone (ITZ)^2–5^. A study on shear bond strength of two-part AAMs by^2^ using a slant shear test suggested that the combination of fly ash and Portland cement improved the bonding strength of AAMs mortar and PCC substrates and recorded slightly above 25 MPa, comparable to the bonding strength that applied with the commercial repair materials. The developed bond strength in two-part AAMs mortar’s trend is further supported by the findings from^5^ on the combination of FA/GGBFS precursors exhibiting higher interfacial adhesion level as a result of conducting an interfacial flexural-tensile strength contributed to the better compressive strength of about 60 MPa and flexural strength of about 6 MPa at 28 days of age.

However, for structural concrete repair materials Class R3 and Class R4, the pull-off bonding strength test method is required and must exceed 1.5 MPa for Class R3 and 2.0 MPa for Class R4 as per EN1504-3 Standard^6^. Salazar et al.^7^ reported that the two-part AAMs mortar composed of 70% natural volcanic pozzolan and 30% ground granulated blast furnace slag (GGBFS), activated with sodium hydroxide (NaOH) and sodium silicate (Na_2_SiO_3_), only recorded 1.24 MPa despite highest compressive strength at 28 days of age about 65 MPa. Two-part alkali-activated mortar composed of a sole slag precursor collapsed when the patch was applied vertical and horizontally due to low pull-off bonding strength between repair materials and substrate. When the slag precursors were substituted and composed of Fly Ash (FA) only, the adhesion strength was recorded 2.3 MPa at the vertical surface and 1.8 MPa horizontally. The two-part AAMs mortar activated by metakaolin-only, however, recorded consistent pull-off bonding strength reading at 2.0 MPa, both tested at vertical and horizontal surfaces. It is worth mentioning that the alkali activator reported in this experiment was between 8 and 14% weightage from the precursor's total weight and demonstrated the influence of the alkali activator where bonding strength will increase when the amount of Si and Na is boosted^6^. Too much alkali content, however, will cost more and affect the mechanical strength with efflorescent, especially metakaolin-based cementitious^8^. Nunes et al.^9^ explained that the precursors composed with GGBFS/ metakaolin under high alkalinity conditions tend to reduce adhesive strength due to the unreactive slag caused by high reactivity calcium precipitation that caused Ca(OH)_2_ drops in the loss of C–S–H type of gels, risking the bond. The average pull-off bond strength recorded reported in this experiment showed that sole metakaolin precursor activated with a SiO_2_/Al_2_O_3_ molar ratio of 3.0 for alkali solution only achieved 1.78 MPa at 28 days of age and 1.74 MPa for precursors activated with 80% GGBFS & 20% metakaolin at a similar molar ratio of alkali solution. It is worth noting that both mortar samples recorded higher compressive strength of 50 MPa at 28 days of age.

In addition, the setting time of the one-part AAMs mortar is too fast if containing a higher percentage of OPC, as reported by^10^, where one-part geopolymer paste recorded 22 min for an initial setting time even though it has remarkable concrete mechanical strength at 7 days of age at around 27 MPa with the inclusion of 60% of OPC, nevertheless, accelerate setting time does not favour the production and handling process at the construction site. Therefore, to overcome the shortcomings of these alternative concrete repair materials, attention should be given more to the mix composition of one-part alkali-activated mortar, particularly on the selection of raw materials as aluminosilicates precursors, type of solid alkali activator, admixtures and the ratio of water and aggregates in the mixtures.

As for the solid alkali activator, the sodium metasilicate series has recorded the shortest setting time for a one-part alkali activator mortar. Increasing the alkali activator amount has a linear growth in compressive strength, for which the flexural strength will follow the same trend. Regular concrete's required initial setting time is more than 45–75 min depending on the strength class^11^. Liu et al.^12^ used honeycomb ceramic (HCC) as a carrier for the alkaline activator to prolong the setting time of one-part alkali-activated slag paste to counter from short setting time of one-part AAMs. By taking into account, the lowest dosage of alkali activator used for one-part alkali-activated slag mortar in the experiment by Almakhadmeh et al.^13^, colder mixing water temperature at 0 ℃ recorded a longer initial setting time, followed by 10 and 20 ℃, whereby 30 ℃ of mixing water temperature shortened the initial setting time up to 30%, or the average of 50–60 min dropped for every 10 ℃ of mixing water temperature difference compared to mortar samples activated with 0 ℃ of water which explained the influence of mixing water temperature in controlling the initial setting time of fresh one-part AAMs.

Askarian et al.^10^ reported that one-part alkali-activated paste composed of fly ash and slag only did not set within the first 24 h. With the inclusion of ordinary Portland cement (OPC), the initial and final setting time for fresh paste was significantly decreased. The superplasticizer (SP) effect on one-part alkali-activated slag mortar was studied by Luukkonen et al.^14^ and found that the lignosulfonate-based superplasticizer was the most suitable retarder admixture to lengthen the setting time of the AAMs slag mortar by increasing the SP and water amount. The delay in setting time was due to adsorption activities by the SP towards binder particles under an alkaline environment^15^. Coppola et al.^16^ reported the influence of admixtures in one-part alkali-activated slag where the calcium oxide, CaO used as an expansive agent for the paste samples reduced both initial and final setting time and the combination of CaO and shrinkage reducing admixture (SRA) effectively decreased shrinkage level of the mortar samples.

Wang et al.^5^ reported that three systems were applicable for the compatibility between repair material and its substrate: mechanical interlocking, Van de Waals forces, and chemical bonds. Teixeira et al.^17^ explained the requirement for repair mortars according to EN 1504 standard used in Europe and highlighted the importance of AAMs properties in fresh and hardened states for sustainable repair and reinforcement elements.

Study on one-part AAMs has been conducted extensively at the synthesis stage to determine the new kind of by-products, chemical activator, and compositions. However, the usage of one-part AAMs as concrete repair materials have not been studied profoundly. Nevertheless, One-part AAMs can be used in concrete and are helpful as mortar. In this experiment, a one-part alkali-activated mortar was carried out to establish its potential as a concrete repair material in terms of compressive strength and also the mortar’s bonding strength which was evaluated by pull-off bonding strength method against OPC substrate to satisfy class R3 and Class R4–EN1504-3 specifications for structural concrete repair materials. In addition, the volume ratio for aluminosilicate precursors was kept constant (25% FA, 5% GGBFS and 7% OPC). The main objective of this paper is to determine the capability of the one-part alkali-activated mortar used as a patching mortar as an alternative concrete repair material product for structural repair purposes class R3 and R4 EN1504-3 specifications.

## Materials and method

Class f–Fly Ash (FA) and Ground Granulated Blast Furnace Slag (GGBFS) were used as precursors under ASTM C618 and ASTM C989, respectively. Table 1 shows the chemical composition of Ordinary Portland cement (OPC), which was added as the primary binder source and activated with alkali-activated powder-potassium carbonate (K_2_CO_3_ Purity ≥ 90%). Natural sand was used as fine aggregates with a specific gravity of 2.67 and an average particle size of 90.23 μm (D50). In addition, a commercial ethylene glycol type of shrinkage reducing admixtures (SRA) and calcium oxide (CaO) was added as admixture and expansion agent, respectively, in the form of solid powder. At the same time, the sodium lignosulfonate powder-based superplasticizer (SP) was also used as a water reducer in high-strength concrete.

**Table 1:** Chemical compositions (%) of Ordinary portland cement (OPC) obtained from the cement manufacturer.

Chemical compositions (%)				
Chemical compositions (%) OPC	CaO	SiO_2_	Fe_2_O_3_	MgO
	60–65%	17–25%	0.5–6.0%	0.1–4.0%
	Al_2_O_3_	Na_2_O + K_2_O	SO_3_	
	3–8%	0.2–1.0%	1.0–2.75%	

### Mix Proportions

The experimental study was conducted to understand the effect of aluminosilicate precursor and OPC as the main binder, activated with a different low dosage of alkali activator powder. Adjusted water content to improve the mortar workability, compressive strength, and bonding strength for patch mortar application and meet the Class R3, EN-1504 standard for structural concrete repair material.

All the samples were marked as Mix 1 to Mix 7 and consisted of FA, GGBFS and OPC as main precursors with different volume percentages. The admixtures proportion for every sample was added into the mortar samples between 1.0 to 15.0 wt% of weight based on total aluminosilicate precursors (binder) weight. The water to binder ratio ranged from 0.30 to 0.50, and the binder to sand ratio was constant at 1 to 1 to produce the mortar applied to all mixtures. The compositions of one-part alkali-activated mortars are further elucidated in Table 2.

**Table 2:** Mix design of one-part alkali-activated repair mortars.

Samples	Binder			Alkali activated	Admixtures			Design ratio	
	FA (%)	GGBFS (%)	OPC (%)	K_2_CO_3_ (%)	SRA (%)	CaO (%)	SP (%)	Binder to sand	Binder to water
*Mix 1*	25	5	70	6	2	1	1	1	0.49
*Mix 2*	25	5	70	1.8	–	–	–	1	0.50
*Mix 3*	25	5	70	1.6	0.3	0.15	1	1	0.40
*Mix 4*	25	5	70	2.0	0.9	0.45	1	1	0.40
*Mix 5*	25	5	70	1.8	0.6	0.30	1	1	0.40
*Mix 6*	25	5	70	1.6	0.3	0.15	1	1	0.35
*Mix 7*	25	5	70	1.6	0.3	0.15	1	1	0.30

### Sample preparations

An electric mixer, EX-EM2000 EXTRAMAN 2000 W, was used to prepare all mixes. The FA, GGBFS, PCC, K_2_CO_3,_ SRA, CaO, Sodium Lignosulfonate (SP) and fine aggregates were blended in the mixture for 2 min according to their sample of mix compositions. After that, water was added slowly to the mixtures and continued blending for another 3 min to ensure the mortar paste was uniform. Then, all the fresh mortars were immediately cast into a 50 mmx50 mmx50 mm cube for the compression strength test. All filled moulds were vibrated for 2 min using a shaking table. The mixtures were demoulded after 24 h before being cured at an ambient lab temperature of 29 ℃, with Relative Humidity (RH) of 65% until the testing day on 7, 14 and 28 days of curing age.

**Figure 1:**
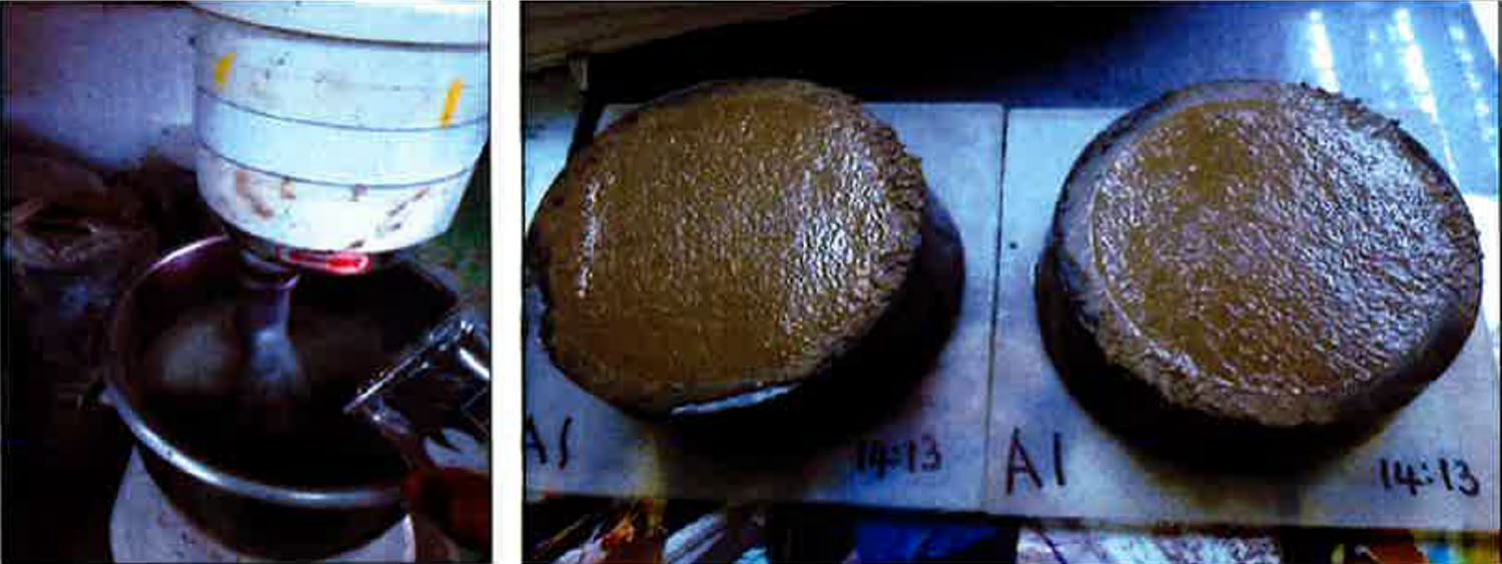
Vicat penetration test for setting one-part alkali-activated repair mortar.

For setting time test of fresh mortar, it follows the same procedure. Still, after 3 min of mixing with water, the samples are immediately cast into Vicat moulds (Fig. 1) before the penetrating process begins at a specific time interval. The preparation of fresh mortar for setting time and pull-off strength test was under a controlled temperature of 21 ℃ and Relative Humidity (RH) > 90%. The age of the concrete substrate is 56 days, and the age of the test specimen is 28 days after curing age.

### Experimental procedures

The one-part AAMs mortar was mixed following the dry mixing method in previous studies. The compressive strength of hardened mortar was evaluated at 7-d, 14-d, and 28-d curing age to study mechanical strength and properties. The compression test machine AUTOMAX5 was used at a loading rate of 1000 N/s. The mean value of three readings of each sample produced in triplicate for every test was recorded and taken as their final strength value. Test on the setting time of mortar was conducted by penetrating the fresh mortar in Vicat moulds, set with 120 min for its first interval. The height of the Vicat mould is 40 +/− 0.2 mm, and the initial setting time was recorded when the 34 +/− 3 mm was obtained, and the final setting time was recorded at 0.5 mm penetration depth obtained as per MS EN 196–3: 2016 specification. A 50 mm dolly size was used for the pull-off strength test, and the core drill depth was 15 mm into the substrate material as per BS EN 1542:1999 requirement.

## Result and discussion

### Setting time of fresh mortar

**Figure 2:**
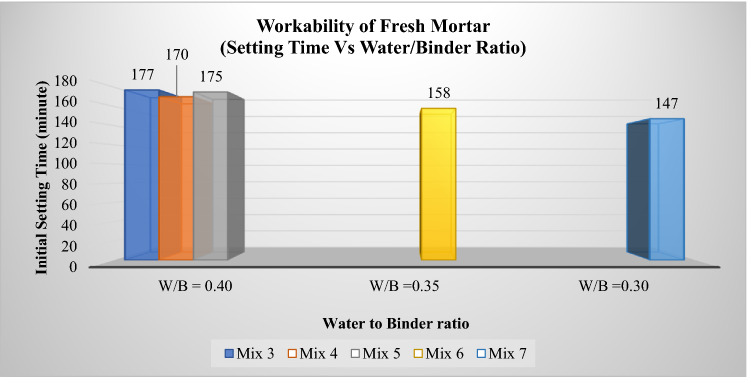
Initial time for a one-part alkali-activated repair mortar (Setting time Vs. Water/Binder ratio).

The initial setting time for the one-part alkali-activated mortar is essential for in situ application. The initial setting time is when fresh mortar starts losing its plasticity and the final setting time is when the mortar completely loses its plasticity. Too long of an initial setting time will cause mortar or concrete to lose strength. This parameter will control the handling process from mixing to the casting stage. The initial setting time for Mix 2 (control sample) was 243 min or 4 h compared with Mix 7, which recorded 147 min, a 39% reduction in initial setting time as shown in Table 3. The result also met the previous report's findings on the initial setting time range for one-part AAMs between 23 and 150 min^11^. Higher water to binder ratios for Mix 1 and Mix 2 affect the overall setting time of the fresh mortar, where the initial setting time was recorded at 320 min and 243 min, respectively. When the water content is reduced to a 0.40 water-binder ratio, both initial and final settings for Mix 3, Mix 4 and Mix 5 decrease in the range of 177 min to 170 min for the initial setting time and between 302 to 289 min or about 5 h for the final setting time. The fastest initial setting time was recorded for sample Mix 7 at 147 min, followed by sample Mix 6 at 158 min, where both samples contained low water-to-binder ratios of 0.30 and 0.35. As illustrated in Fig. 2, the overall pattern of initial setting time decreased against lower water (W/B) content levels.

**Table 3:** Initial and final setting time of one-part alkali-activated repair fresh mortar.

Mix samples	Initial setting time (min)	Final setting time (min)
*Mix 1*	320	401
*Mix 2 (control sample)*	243	319
*Mix 3*	177	302
*Mix 4*	170	289
*Mix 5*	175	295
*Mix 6*	158	279
*Mix 7*	147	274

**Figure 3:**
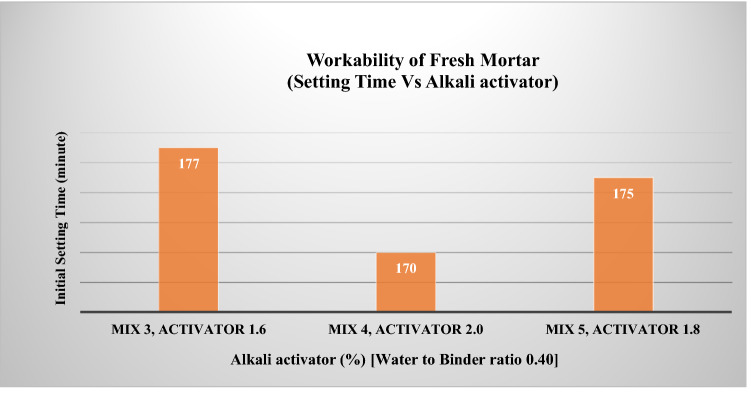
Initial setting time for a one-part alkali-activated repair mortar (Setting time Vs. Alkali activator).

It was reported that the common problem with one-part AAMs was they were set too fast and had a typical initial setting time in the range of 60 min, as reported by^18^. The longer setting time recorded in this experiment agrees with^12^ that the presence of slag and solid potassium carbonate could reduce the workability of a one-part geopolymer. As shown in Fig. 3, three mortar samples Mix 3, Mix 4 and Mix 5, have similar water to binder ratios of 0.40. Still, the longer initial setting time for Mix 3 was mainly contributed by the low alkali activator dosage of potassium carbonate, which only consists of 1.6% of total precursors weight because the higher the activator dosage, the shorter the setting time of one-part AAMs. Furthermore, all samples were prepared under a colder controlled temperature of 21 ℃, which could be why lengthens the initial setting time due to the thermal effect factor as in the agreement with^13^. Furthermore, 70% OPC content used as primary aluminosilicate sources for the binder was also the possible reason for a longer initial setting time due to a large group of cement particles occurring when the former reacts with water for the hardening process subsequently increased yield stress and plastic viscosity of the mortar for a longer setting time^19^.

Potassium carbonate powder used in this experiment is a chemically non-hygroscopic alkaline type and reported to have a minimal impact than typical hygroscopic-based activators such as sodium hydroxide and sodium silicate that could harm the product in terms of efflorescence and water absorption level^10^. However, the slower dissolution of solid potassium carbonate activators, especially K ions in water, caused a slow hardening process at an early stage^20^. This experiment also confirmed the additional 1% (of binder weight) lignosulfonate-superplasticizer (SP) and higher water content could prolong the setting time of one-part alkali-activated mortar reported by^14^. Activated by a constant low dosage of alkali activator in resulting longer initial setting time of more than 100 min for both mortar samples respectively, the difference composition between the control samples Mix 2 and Mix 3 was that the addition of SRA and CaO provided more calcium to Mix 3 for a rapid reaction at an early stage on top of the existing 70% OPC for all mortar samples Mix 1 to Mix 7. Thus, SP was added to control the setting time of all samples with rich calcium examined in this experiment.

Comparing the initial setting time between Mix 2 (without admixtures) and Mix 1, 3–7, the strong influence of admixtures can shorten the setting time and improve the mechanical strength of one-part alkali-activated mortar. However, without admixtures, the control sample Mix 2 has recorded a longer initial setting time and low compressive strength at 28 days of curing age and does not comply with class R3 EN1504-3 standard.

It is worth noting that the non-hygroscopic alkaline with low alkali activator content of 1.6% was used in this experiment yet achieved not only mechanical strength comparable to the one-part AAMs activated by a conventional higher dosage of hygroscopic activator type but also a more extended initial setting for the mortar to become not too short or not too long and improved the setting time issues on one-part AAMs system.

### Compressive strength

Table 4 above shows the compressive strength of one-part alkali-activated mortar composed of mixed ordinary Portland cement (OPC), by-products of fly ash and ground granulated blast furnace slag (GGBFS). All samples were activated by a low alkaline activator which is not corrosive, greener, yet cheaper. Mix 3 was referred to as a control sample based on the workability result activated by the lowest dosage of alkali activator at water to binder ratio of 0.40. At the same time, Mix 2 was composed without admixtures.

**Table 4:** Compressive strength at 7, 14 and 28 days of curing age.

Sample references	Compressive strength at 7 days (N/mm^2^)	Compressive strength at 14 days (N/mm^2^)	Compressive strength at 28 days (N/mm^2^)
*Mix 1*	19.22	20.92	26.35
*Mix 2*	18.16	17.50	9.26
*Mix 3 (control sample)*	23.76	22.41	26.75
*Mix 4*	19.16	19.03	14.41
*Mix 5*	18.0	18.83	13.88
*Mix 6*	21.90	29.24	36.27
*Mix 7*	21.45	31.14	36.80

At the early stage, the highest compressive strength was recorded for Mix 3 with 23.76 N/mm^2^, followed by sample Mix 6 and sample Mix 7, 21.90 N/mm^2^ and 21.45 N/mm^2^, respectively. It is understood that one-part AAMs have a rapid reaction at an early stage due to the dissolution process of solid alkali activator with the presence of rich-calcium oxide content in precursors. The least amount of slag in this experiment, however, has been compensated by adding calcium oxide (CaO) powder as an expansive agent^16^ together with the higher volume of calcium-rich OPC to provide more calcium, subsequently offering additional nucleation sites for dissolved materials under rapid dissolution process at an early stage, contributed to the fast hardening^10,21^.

At 14 days of curing age, samples Mix 1, Mix 5, Mix 6, and Mix 7 recorded an increment in strength, contrary to samples Mix 2 (without admixtures), Mix 3 (control sample) and Mix 4, which recorded slightly decreased strength over time. Mix 7 recorded the highest compressive strength with 31.14 N/mm^2^, followed by Mix 6 with 29.24 N/mm^2^ and Mix 5 with 22.42 N/mm^2^.

Further examination of the mortar’s mechanical strength later confirmed the strong growth over samples Mix 1, Mix 3, Mix 6, and Mix 7. The highest compressive strength recorded was 36.80 N/mm^2^ for Mix 7. The compressive strength slightly dropped for Mix 6 with 36.27 N/mm^2^, followed by Mix 3 and Mix 1, which recorded not much difference in the strength, 26.75 N/mm^2^ for the former and 26.35 N/mm^2^ for the latter. The compressive strength at 28 days of curing age for all four repair mortar samples also complied with a minimum requirement for class R3, EN1504-3 specification for concrete structural repair materials. On the other hand, Mix 2, Mix 4, and Mix 5 continue suffered strength inclination between 30 and 50% over time.

The early mechanical strength of one-part AAMs contributes to the fast reaction due to the rapid dissolution of solid alkali activator in the binder to generate heat and make them lose their plasticity earlier than the two-part AAMs system. Consistent compressive strength levels in the range of 18 N/mm^2^ to 21 N/mm^2^ recorded at an early stage proved the rapid geopolymerization process begins immediately after water is added. Samples Mix 3, Mix 4, and Mix 5 were composed with a low alkaline activator (below 2.0% dosage). In contrast, sample Mix 2 activated without admixtures caused low compressive strength compared with the samples containing added admixtures. Samples Mix 4 and Mix 5 mortar have a double and triple volume of SRA and CaO compared to the sample Mix 3 mortar, where SP dosage was kept constant at 1% for all samples. Samples Mix 1 – Mix 5 had compressive strength rise and dropped between − 5% to + 10% within 7 days to 14 days of age, in contrast with common hardened mortar, which is usually springing up to its strength and achieve 90% compressive strength at 14 days of curing age.

The reduction in activator concentration would decrease the mechanical strength of hardened one-part AAMs^22^; thus, the dosage of alkali activator increased to 1.8% and 2.0% for samples Mix 4 and Mix 5 and additional Ca was obtained from SRA and CaO powder. However, the trend showed that dissolution of alkali activator for both samples Mix 4 and Mix 5 reached its peak at 7 days, whereby the lack of alkali activator did not make sufficient to react with the excessive amount of calcium resulting in incomplete reaction and inadequate binding between precursor and aggregate, creating more pores and crack propagation prevent the compressive strength growth at a later stage. Also, declined compressive strength over time was found in the fact that one-part AAMs were not water-resistant compared to the two-part AAMs system and subsequently encountered a slow hydration rate^8^, associated with a higher water/binder ratio of 0.4 to 0.5, in addition to the low dosage of alkali activator used to compose these mortar samples.

Moreover, samples Mix 6 and Mix 7 recorded the highest compressive strength, about 36 N/mm^2^, which is higher than the 25 N/mm^2^ set as minimum compressive strength at 28 days of age for class R3-EN1504-3 specifications. Both mortar samples have similar mix compositions except for the water/binder ratio, 0.3 for Mix 7 and 0.35 for Mix 6.

Likewise, it is interesting to know that the low dosage of alkali activator has activated both mortar samples with 1.6% only than sample Mix 1 mortar activated by 6% yet achieved consistent and higher compressive strength. Insufficient Si and Al ions would dissolve due to a low alkalinity environment, and the resulting reduction in aluminosilicate precursors subsequently affects the rates of geopolymeric reaction^23^. On the other hand, Coppola et al.^24^ reported that a higher dosage of alkali activator is required to lower the water demand. The lower alkaline activator incorporated with lower water to binder ratio contributed to the higher compressive strength. Li et al.^19^ however, explaining the effect of the water to binder ratio plays a vital role in determining the rheological parameters of a one-part AAMs system. A lower w/b ratio will significantly increase yield stress and plastic viscosity for one-part AAMs and ordinary Portland cement (OPC). Luukkonen et al.^25^ claimed that less water will increase the compressive strength of one-part AAMs, and adding a superplasticizer can further reduce and control the water. This experiment also confirmed that the presence of CaO and SRA restricted the growth of mechanical strength of one-part alkali-activated repair mortar as in the agreement with Coppola et al.^16^

### Pull-off bonding strength

**Figure 4:**
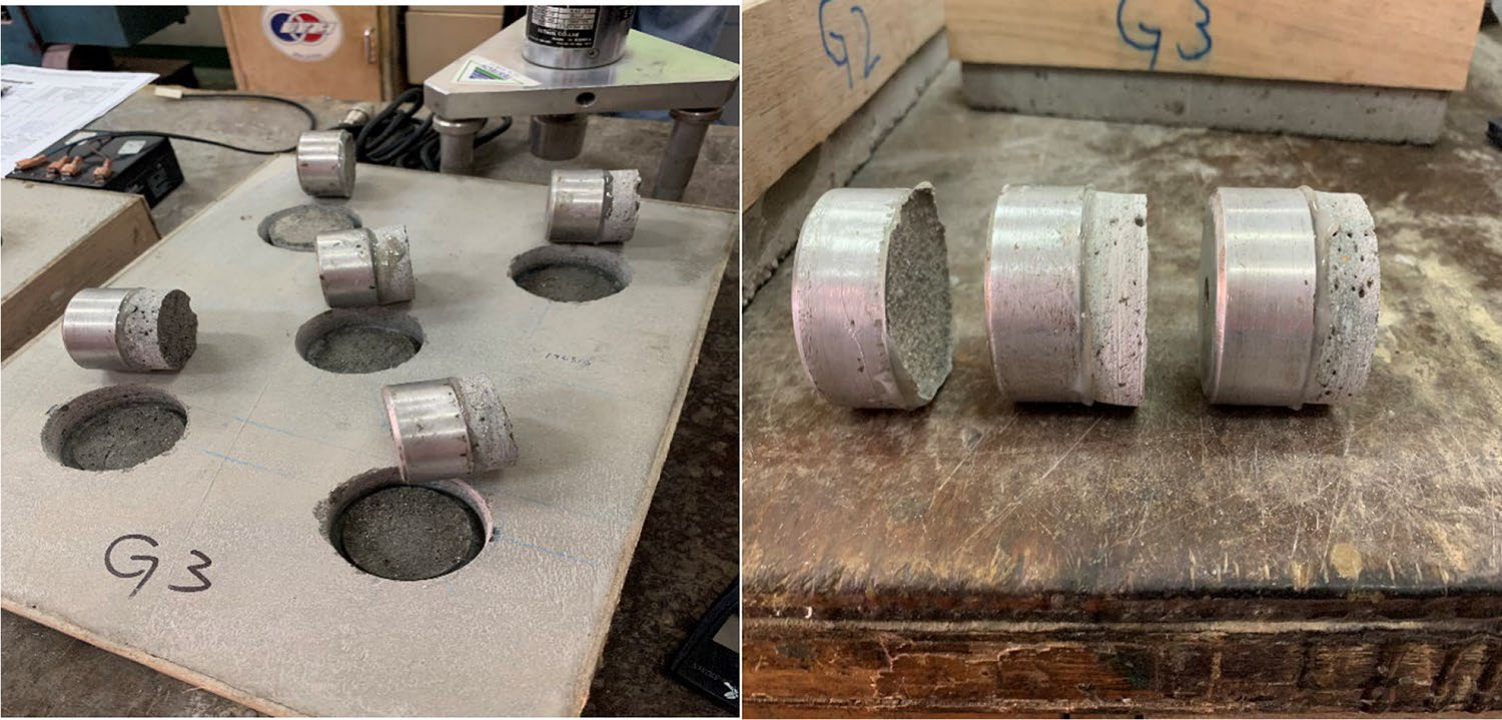
Pull off mortar samples after 28 days of age (Mix 7).

Based on the compressive strength result shown in Table 4, which was recorded above 25 MPa, only 3 samples were selected to be tested with compression. Pull-off bond strength for all samples complied with the required strength requirement Class R3–EN1504-3 standard for structural concrete repair materials. The highest pull-off bond strength was recorded for sample Mix 7 mortar with 2.565 MPa (Class R4–EN504-3 standard), Mix 3 with 1.885 MPa and Mix 6 with 1.757 MPa, as shown in Table 5 above. This experiment's pull-off test also confirmed the adhesion mode of failure at the interface between mortar and concrete substrate for Mix 3 and Mix 6, a cohesion mode of failure for Mix 7 (Fig. 4). Both types of failure are considered ideal types of failure classification for the pull-off test as per EN1542 standard, indicating the one-part AAMs repair mortar as a good bonding material. In addition, the higher pull-off bond strength recorded indicates that this type of mortar can provide higher adhesive force^23^. The variety of material compositions sought for mortar mix design in this experiment influences the bond strength of the one-part AAMs system^26^.

**Table 5:** Average pull-off bond strength results in protection and repair products of concrete structures at 28-d of curing age of one-part alkali-activated repair mortar.

Samples references	Tensile bond strength (MPa)	Mode of failure
*Mix 3 (control samples)*	1.885	A and B interface/Adhesion
*Mix 6*	1.757	A and B interface/Adhesion
*Mix 7*	2.565	A–substrate/Cohesion

The reaction between alumina and silica from FA and calcium ions from ordinary Portland cement (OPC) could form C–(A)–S–H gel co-existed with N–A–S–H, enhancing the alkali-activated materials (AAMs) properties as reported by Phoo-ngernkham et al.^2^. The surface of OPC substrates is rich with calcium hydroxide (Ca (OH)_2_) will bond chemically by reacting with the alkaline one-part alkali-activated repair mortar for positive ions such as Ca^++^ to balance the negative charge of the Al^3+^ and Si^4+^ from FA and GGBFS for geopolymeric reaction, in addition of the formation of calcium carbonate hydrate from the reaction between potassium carbonate powder and calcium hydroxide of OPC later improved the adherence between repair mortar and substrates^7,27^. Additional C–S–H and/or C–A–S–H gel was established with N–A–S–H gel enhancing the bonding concentration at the contact area^28^.

This experiment proved that one-part alkali-activated repair mortar, known as the third mortar category, can be used for concrete structural repair to replace conventional organic and inorganic binders in the construction sector^29^. Once again, the low dosage of alkaline activator used in this experiment successfully activated the one-part AAMs system and performed better than the two-part AAMs system counterpart.

## Conclusions

The experiments in this report were carried out to investigate the mechanical properties of compressive and the pull-off bonding strength of one-part alkali-activated mortar against OPC substrate used as concrete patching materials as stated in EN1504-3 guideline for structural concrete repair material specifications. Hybrid precursors of fly ash, ground granulated blast furnace slag and ordinary Portland cement (OPC) were used as the source of aluminosilicate binder. A low alkali activator of potassium carbonate in powder form was used to initiate the geopolymerization process and further adjusted with some admixtures, water, and aggregates for optimum mortar mix design. To maintain workability, a longer setting time is essential for transporting a large quantity of mortar to the construction site. The initial setting time for the mortar sample in this experiment achieved 147 min, in line with previous reports on the setting time of one-part AAMs. In addition, the highest compressive strength and bonding strength at 28 days of age were recorded for Mix 7 with 36.8 N/mm^2^ and 2.565 MPa, respectively**.** Therefore, they complied with Class R4 – EN1504 for structural concrete repair materials.

Another conclusion that can be highlighted in this paper is as follow:

1. Two-part AAMs composed of a single precursor such as fly ash, slag or metakaolin only have higher early compressive strength and better mechanical properties but are not suitable for concrete repair material due to lower pull-off bonding strength. In addition, two-part AAMs mortar activating with sole precursors also does not comply with mechanical requirements for repair materials (pull-off strength class R3 and R4, EN1504 standard) and is often delaminated. However, all these industrial wastes can be utilized by incorporating Portland cement in one-part AAMs technology to promote better chemical bonding between repair mortar and concrete substrates at the interface zone in terms of pull-off bonding strength.
2. A short initial setting time for AAMs mortar is the main problem for supplying process at the construction site. Therefore, a longer initial setting time for the one-part alkali-activated repair mortar recorded in this experiment is beneficial for the in-situ application. The main aluminosilicate precursors of mortar sample Mix 7 were 25% FA, 5% GGBFS and 70% OPC. They exhibited 147 min for the initial setting time within the range of 23–150 min as reported for the initial setting time of one-part AAMs. Furthermore, it is worth noting that countries with hot and dry climates possess a higher rate of hydration. This result enlightened the sustainability of this type of repair mortar which has a flexible range of initial to final setting time when exposed to the higher surrounding temperature that may shorten the setting time of the fresh mortar up to a 30% reduction. Therefore, it is applicable to be applied in hot climate countries.
3. A lower dosage of solid admixtures is used to enhance the fresh and mechanical properties of one-part alkali-activated mortar that meet the requirement for class R3 and class R4, EN1504-3 specifications for structural concrete repair materials together with the optimum admixture dosage of 0.3% for SRA, 0.15% for CaO and 1% of SP from the total weight of precursors. In addition, lower water to binder ratio at 0.30 has increased the repair mortar's pull-off bonding strength following higher compressive strength recorded at 28 days of curing age.
4. The repair mortar assessed in this experiment is composed of fly ash and slag as part of the mix composition, whereby this type of by-product can be developed further to enhance its potential and exploited as cement binder used as concrete patching materials, subsequently overcoming the limitations on the pull-off bonding strength properties as the weakest part of the system as reported. Therefore, future studies should focus on the microstructure analysis of the one-part AAMs mortar to enhance their potential to be used as a concrete repair material.

## Data Availability

The datasets used and/or analyzed during the current study are available from the corresponding author upon reasonable request.
